# Resveratrol Analog 4-Bromo-Resveratrol Inhibits Gastric Cancer Stemness through the SIRT3-c-Jun N-Terminal Kinase Signaling Pathway

**DOI:** 10.3390/cimb44010005

**Published:** 2021-12-22

**Authors:** Yun-Shen Tai, Yi-Shih Ma, Chun-Lin Chen, Hsin-Yi Tsai, Chin-Chuan Tsai, Meng-Chieh Wu, Chih-Yi Chen, Ming-Wei Lin

**Affiliations:** 1 Department of Surgery, China Medical University, An-Nan Hospital, Tainan 70965, Taiwan; taiyskimo@gmail.com; 2 Department of Surgery, E-Da Hospital, Kaohsiung 82445, Taiwan; 3 Institute of Medicine, Chung Shan Medical University, Taichung 40201, Taiwan; 4 Department of Chinese Medicine, E-Da Hospital/E-Da Cancer Hospital/E-Da Dachang Hospital, Kaohsiung 82445, Taiwan; m2367591@ms25.hinet.net (Y.-S.M.); ed103622@edah.org.tw (C.-C.T.); 5 The School of Chinese Medicine for Post-Baccalaureate, I-Shou University, Kaohsiung 82445, Taiwan; 6 Department of Biological Science, National Sun Yat-sen University, Kaohsiung 80424, Taiwan; chun-linchen@mail.nsysu.edu.tw; 7 Department of Medical Research, E-Da Hospital/E-Da Cancer Hospital, Kaohsiung 82445, Taiwan; y7952pipi@gmail.com; 8 Department of Internal Medicine, Kaohsiung Municipal Ta-Tung Hospital, Kaohsiung 80145, Taiwan; 930293@mail.kmuh.org.tw; 9 Department of Nursing, College of Medicine, I-Shou University, Kaohsiung 82445, Taiwan

**Keywords:** 4-bromo-resveratrol, gastric cancer, cancer stemness, chemosensitivity

## Abstract

Chemotherapy is the treatment of choice for gastric cancer, but the currently available therapeutic drugs have limited efficacy. Studies have suggested that gastric cancer stem cells may play a key role in drug resistance in chemotherapy. Therefore, new agents that selectively target gastric cancer stem cells in gastric tumors are urgently required. Sirtuin-3 (SIRT3) is a deacetylase that regulates mitochondrial metabolic homeostasis to maintain stemness in glioma stem cells. Targeting the mitochondrial protein SIRT3 may provide a novel therapeutic option for gastric cancer treatment. However, the mechanism by which stemness is regulated through SIRT3 inhibition in gastric cancer remains unknown. We evaluated the stemness inhibition ability of the SIRT3 inhibitor 4′-bromo-resveratrol (4-BR), an analog of resveratrol in human gastric cancer cells. Our results suggested that 4-BR inhibited gastric cancer cell stemness through the SIRT3-c-Jun N-terminal kinase pathway and may aid in gastric cancer stem-cell–targeted therapy.

## 1. Introduction

Gastric cancer is the most common cause of malignancy and cancer-related mortalities worldwide [1]. Systemic chemotherapy with multiple drugs may be an effective strategy for patients with recurrent gastric cancer. Numerous studies have reported that chemotherapeutic resistance in gastric solid tumors results from genetic heterogeneity in tumor cells. Studies have suggested that cancer stem cells (CSCs) contribute to chemotherapy drug resistance [2]. CSCs are a subpopulation of tumor masses with the ability to self-renew, maintain stemness, and cause cancer recurrence [3]. CSCs may be a novel target for anticancer strategies and gastric cancer therapy in precision medicine [2].

CSCs in gastric cancer were first described in 2007 [4]. The most common way to identify gastric CSCs is through investigating the expression of cell surface markers, including CD24 and LGR5. CD24 is a cell surface protein that is expressed in putative stem cells and is overexpressed in various malignant tumors. It was reported to stimulate metastasis and trigger epithelial-to-mesenchymal transition through Notch1 signaling [5]. Leucine-rich repeat-containing G-protein coupled Receptor 5 (LGR5) is overexpressed in stem cell after *Helicobacter pylori* infection in the gastrointestinal tract [6], which is reported to be able to initiate tumors [7,8], and has been defined as a gastric cancer stem cell marker recently [9,10]. Nanog is a transcription factor widely expressed in human cancers [11,12,13]. It drives self-renewal, metastasis, and chemoresistance [14,15]. The transcription factor Sox2 is involved in the maintenance of an undifferentiated cellular phenotype [16]. Its aberrant expression in cancers often leads to increased chemotherapy resistance [17]. Oct-4 is also a transcriptional factor to regulate pluripotency in the mammalian stem cell population. It is upregulated in several cancers and promotes chemoresistance, metastasis, and recurrence [18,19,20]. Overexpression of these transcription factors, Nanog and Oct-4, promoted the formation of tumor spheroids in vitro [21], and correlated to a poor prognosis of the patient [22,23,24]. The other functional marker, aldehyde dehydrogenase 1 (ALDH1) is widely used to characterize cancer stemness in many studies [25,26,27]. ALDH1 is a detoxifying enzyme responsible for oxidation. Elevation of its activity may protect CSCs against cell death caused by ROS [28]. Increased ALDH1 activity has been found in malignant cancer cells, and served as an indicator for poor prognosis [29].

Sirtuin-3 (SIRT3) is a deacetylase in mitochondria. SIRT3 regulates the mitochondrial respiratory capacity and reduces production of reactive oxygen species, facilitating adaptation to stress. In glioma stem cells, SIRT3 was reported to regulate glioma CSCs with metabolic plasticity and maintain cancer stemness [30]. Its enzyme activity increases chemoresistance. Inactivation of SIRT3 leads to metabolic alterations, loss of stemness, and suppression of tumor formation by CSCs [31]. Ma et al. demonstrated that SIRT3 contributes to chemoresistance through mitochondrial metabolism reprograming and reactive oxygen species downgeneration [32]. SIRT3 is associated with enhanced gastric cancer risk and can thus be a potential prognostic marker [33]. However, the inhibition of SIRT3 activity involved in gastric cancer stemness and chemoresistance has not been thoroughly explored.

The resveratrol analog 4′-bromo-resveratrol (4-BR) has been reported to inhibit SIRT3 activity considerably [34] and suppress melanoma cell growth through mitochondrial metabolic regulation [35]. We evaluated the effects of 4-BR on cancer stemness and chemoresistance inhibition in gastric cancer cells. We postulated that 4-BR would use the SIRT3-mediated pathway to downregulate gastric cancer stemness and increase chemosensitivity.

## 2. Materials and Methods

### 2.1. Cell Culture and Reagents

Human gastric cancer cell lines MKN45 (DSMZ; ACC-409) and AGS (ATCC; CRL-1739) were purchased from DSMZ (Braunschweig, Germany) and ATCC (Manassas, VA, USA), respectively, and cultured using RPMI1640 (Gibco, Waltham, MA, USA) medium with 10% fetal bovine serum (Gibco, Waltham, MA, USA) under 5% CO_2_ at 37 °C. The 4-BR, SP600125, and 5-fluorouracil (5-FU) (Sigma-Aldrich) were purchased from Sigma-Aldrich (St. Louis, MO, USA).

### 2.2. Cell Viability Analysis

Human gastric cancer cells were seeded in 96-well dishes in quadruplicate at 6000 cells/well and cultured for 24 h before treatment. The cell viability of AGS or MKN45 was evaluated with 5-FU (0.5 μM), 4-BR (25 μM), or a combination of 5-FU and 4-BR for 48 h. Cell viability was analyzed using Cell Counting Kit-8 (Sigma–Aldrich, St. Louis, MO, USA), and absorbance was measured at 450 nm by using a microplate reader.

### 2.3. Flow Cytometry Analysis

After treatment, the cells were washed with cold phosphate buffer saline and stained with surface marker CD24 (BD Biosciences, San Jose, CA, USA) before being analyzed through flow cytometry. For the ALDH1 activity evaluation, the cells were stained using the AldeRed ALDH Detection Assay kit (Sigma–Aldrich, St. Louis, MO, USA) and analyzed through flow cytometry.

### 2.4. Western Blotting Analysis

The protein expressions of MKN45 were evaluated through Western blotting after 4-BR (vehicle or 12.5 μM or 25 μM) or JNK inhibitor SP600125 (20 μM) treatment for 48 h. After 4-BR or SP600125 treatment, the human gastric cancer cells were washed with phosphate buffer saline. The total protein samples were extracted, and protein concentrations were measured using Bio-Rad Bradford Protein Assays (Bio-Rad, Hercules, CA, USA). Total protein was separated using sodium dodecyl sulfate–polyacrylamide gel electrophoresis and transferred onto polyvinylidene fluoride membranes. The membranes were incubated with blocking buffer (Bio-Rad, Hercules, CA, USA) for 30 min at room temperature and with primary antibodies: SOX2 (1:1000; #23064; Cell Signaling, Beverly, MA, USA), OCT4(1:1000; #2750; Cell Signaling, Beverly, MA, USA), β-actin (1:5000; #4967; Cell Signaling, Beverly, MA, USA), Notch1 (1:1000; #3608; Cell signaling, Beverly, MA, USA), Sirt3(1:1000; #5490; Cell Signaling, Beverly, MA, USA), JNK (1:1000; #9252; Cell Signaling, Beverly, MA, USA), and p-JNK (1:1000; #9251; Cell Signaling, Beverly, MA, USA) at 4 °C overnight. After washing with phosphate-buffered saline with Tween, the membranes were developed using an enhanced chemiluminescence detection system under MultiGel-21 system (TOP BIO Co., New Taipei City, Taiwan) after incubation with secondary antibodies.

### 2.5. Soft Agar Colony Formation Analysis

For the colony formation assay, MKN45 cells (2.5 × 10^4^ cells) were seeded in 6-well dishes and coated in 0.3% agar. After 2 weeks, the colonies were stained with 0.005% crystal violet, and the area was measured using ImageJ.

### 2.6. Sphere Formation Analysis

For the sphere formation analysis, MKN45 cells (1 × 10^4^ cells) were seeded in a NanoCulture Plate (SCIVAX, Kanagawa, Japan) and incubated with 4-BR for 7 days; the spheres were evaluated using ImageJ.

### 2.7. Statistical Analysis

All data were analyzed using GraphPad Prism (version 8). All graphs in the figures present mean ± standard deviations. For statistical analysis, Student’s *t* test was performed to compare data between the two groups. Statistical significance was set at *p* < 0.05. Statistical results were labeled in each figure as follows: * *p* < 0.05, ** *p* < 0.01, and *** *p* < 0.001.

## 3. Results

### 3.1. 4-BR Reduced Gastric Cancer Viability and Cancer Stemness Capacity

**Figure 1:**
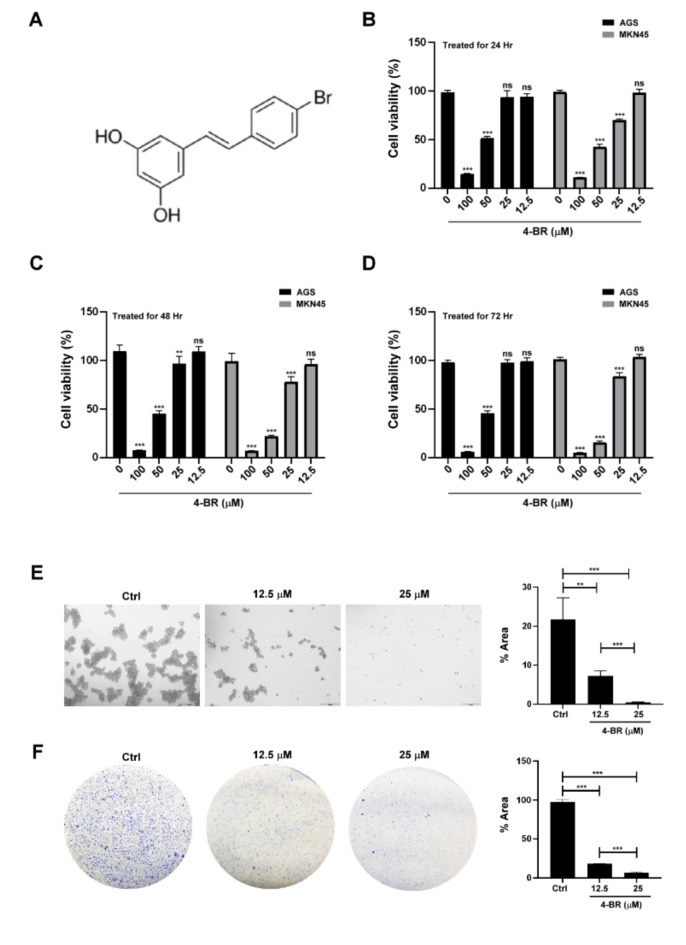
4-BR reduced cell viability and stemness capacity in human gastric cancer cells. (**A**) Structural formula of 4-BR. (**B**) MKN45 and AGS were treated with various doses of 4-BR (0, 12.5, 25, 50, and 100 μM) for 24, (**C**) 48, or (**D**) 72 h. (**E**) The 4-BR treatment limited the sphere formation ability in MKN45 (with light microscopy at ×40). (**F**) The 4-BR treatment suppressed the colony formation ability in MKN45 (with light microscopy at ×10). Data are presented as the means ± standard error; *n* ≥ 3 independent experiments. Student’s *t* test: ** *p* < 0.01, *** *p* < 0.001.

To evaluate the cytotoxicity of 4-BR to human gastric cancer cells, the viability of MKN45 and AGS was evaluated through a Cell Counting Kit 8 assay after the treatment of various doses of 4-BR. The results indicated that 4-BR inhibited gastric cancer growth in a dose-dependent manner (Figure 1B–D). The stemness properties of the human gastric cancer cells were then evaluated using a sphere formation and colony formation assay after the low-cytotoxicity 4-BR (25 μM) treatment. The results indicated that 4-BR inhibited the stemness properties of the MKN45 cells (Figure 1E,F).

### 3.2. 4-BR Downregulated Stemness-Related Protein Expressions in Human Gastric Cancer Cells

**Figure 2:**
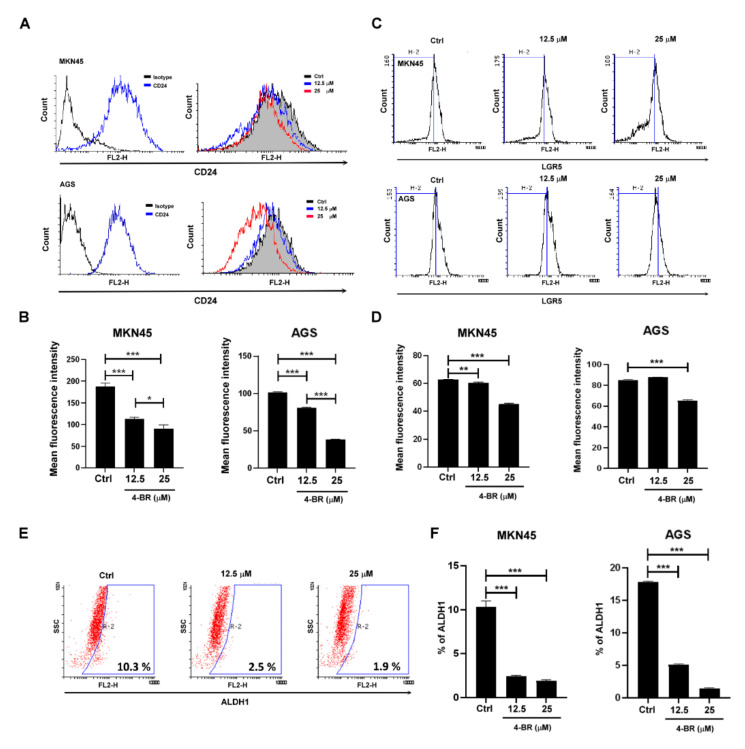
Gastric cancer stemness-related markers were inhibited by 4-BR. (**A**) CD24 expression was analyzed through flow cytometry after 4-BR (12.5 or 25 μM) treatment for 48 h in MKN45 and AGS cells. (**B**) Quantification of CD24 expression. (**C**) LGR5 expression was analyzed through flow cytometry after 4-BR (12.5 or 25 μM) treatment for 48 h in MKN45 and AGS cells. (**D**) Quantification of LGR5 expression. (**E**) ALDH1^+^ cells were analyzed through flow cytometry after 4-BR (12.5 or 25 μM) treatment in MKN45 and AGS cells. (**F**) Quantification of ALDH1^+^ cells. Data are presented as means ± standard error; *n* ≥ 3 independent experiments. Student’s *t* test: * *p* < 0.05, ** *p* < 0.01, *** *p* < 0.001.

**Figure 3:**
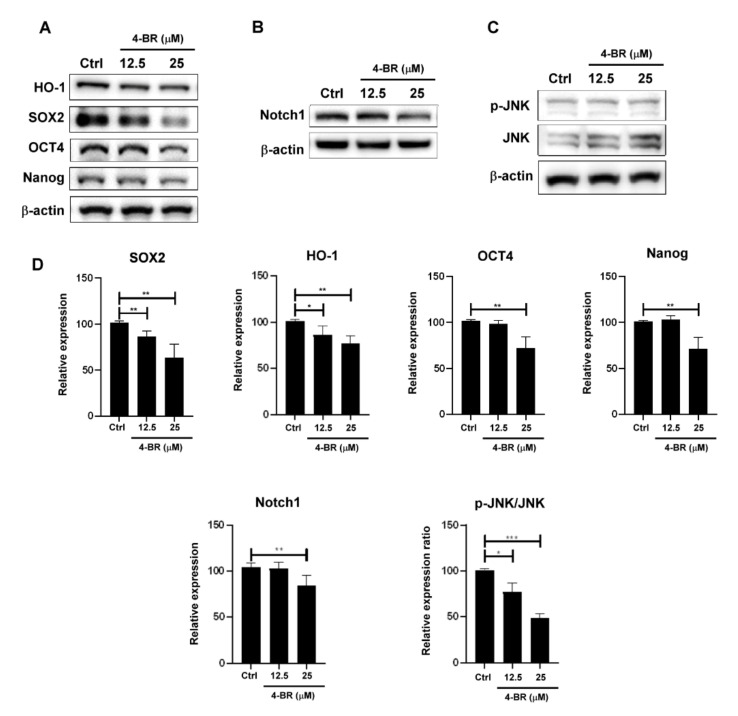
4-BR inhibited cancer stemness-related transcriptional factors in human gastric cancer cells. (**A**) HO-1, cancer stemness-related transcriptional factor proteins, (**B**) Notch1, (**C**) and JNK phosphorylation in MKN45 cells were analyzed through Western blotting after 4-BR treatment in MKN45 cells for 48 h. (**D**) Quantification of protein expressions. Data are presented as means ± standard error; *n* ≥ 3 independent experiments. Student’s *t* test: * *p* < 0.05, ** *p* < 0.01, *** *p* < 0.001.

To evaluate stemness-related protein expression, CD24, LGR5, and ALDH1 activity was measured through flow cytometry (Figure 2), and HO-1, SOX2, Nanog, and Notch1 were evaluated using Western blotting (Figure 3A,B) after a 4-BR (25 μM) treatment for 48 h in MKN45. Activated c-Jun N-terminal kinase (JNK) has been reported to regulate Notch1 expression and the self-renewal of stem cells [36,37]. The results indicated that the 4-BR treatment reduced the expression of markers related to gastric cancer stemness, including Notch, resulting in JNK dephosphorylation and reduced downstream signaling.

### 3.3. 4-BR Contributed to JNK-Mediated Gastric Cancer Stemness Inhibition and Increased Chemosensitivity to 5-FU

**Figure 4:**
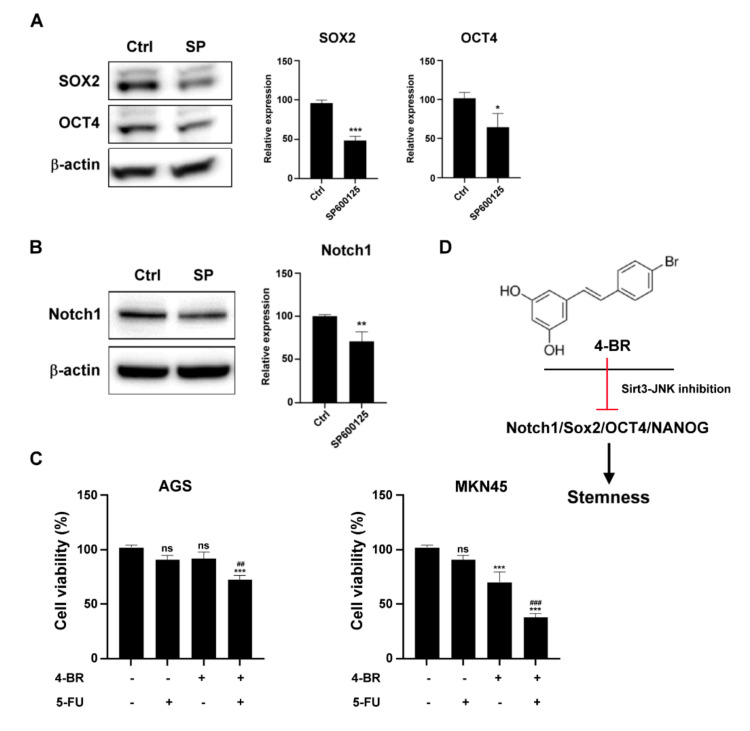
4-BR contributes to the inhibition of JNK-mediated cancer stemness and increases chemosensitivity to 5-FU. (**A**) JNK inhibitor SP600125 (SP; 20 μM) inhibited stemness transcriptional factors and Notch1 in MKN45. (**B**) Quantification of stemness transcriptional factors and Notch1 after JNK inhibitor treatment for 48 h. (**C**) The cell viability of AGS or MKN45 was evaluated through a Cell Counting Kit assay with 5-FU (0.5 μM), 4-BR (25 μM), or a combination of 5-FU and 4-BR. (**D**) Role of 4-BR in gastric cancer stemness inhibition. Data are presented as means ± standard error; *n* ≥ 3 independent experiments. Student’s *t* test: * *p* < 0.05, ** *p* < 0.01, *** *p* < 0.001, ## *p* < 0.01, ### *p* < 0.001.

To determine whether 4-BR contributed to JNK-mediated gastric cancer stemness inhibition, we used the JNK inhibitor SP600125 to evaluate the stemness-related transcriptional factor expression of the human gastric cancer cells. The data revealed (Figure 4) that inhibition of JNK activity reduced SOX2, Oct4, and Notch1 expression. To determine whether 4-BR increased chemosensitivity after the inhibition of cancer stemness properties, combined treatment with 4-BR and the clinical chemotherapy drug 5-FU was administered to MKN45 and AGS cells. The results indicated that 4-BR reduced human gastric cancer cell chemoresistance, suggesting that 4-BR increases chemosensitivity by inhibiting stemness and the SIRT3-JNK pathway.

## 4. Discussion

CSCs are the distinct subpopulation of cells which have the ability to self-renew and are not destroyed by conventional treatments. The failure of anticancer treatment has been attributed to tumor recurrence due to CSCs [38]. Targeting CSCs may prevent or delay tumor development, metastasis chemoresistance, and recurrence. Therefore, it is important to target CSC specific markers for CSCs elimination.

Knockdown of SIRT3 decreases the expression of antioxidant enzymes [39]. Suppression of HO-1 through the knockdown of antioxidant transcription factor nuclear factor increases the sensitivity of 5-FU-resistant pancreatic cancer cells [40]. SIRT3 promotes cellular proliferation and may be involved in 5-FU-induced autophagy, epithelial-mesenchymal transition, and chemoresistance in gastric cancer cells [41]. Our results revealed that inactivation of SIRT3 by 4-BR reduced the expression of the antioxidant enzyme HO-1 and stemness-related proteins and increased chemosensitivity to 5-FU. Aberrant SIRT activity affects metastatic and oncogenic progression. Studies have proposed SIRT3 as a prognostic predictor for breast cancer and ovarian cancer [42,43,44]. SIRT3 may provide a novel therapeutic option for gastric cancer. Synthetic SIRT inhibitors are crucial, and 4-BR is a promising candidate for clinical therapeutic use.

Notch1 signaling is a major cell communication system during organ development and plays a crucial role in oncogenesis and stem regulation of cells [45,46]. It also frequently upregulates in aggressive tumors [47]. The Notch1 pathway controls cancer stem cell proliferation and fate [48,49,50]. Notch1 inhibition may possess potential benefits in clinical cancer therapy [51]. JNKs are members of the mitogen-activated protein kinase family. Activated JNKs catalyze the phosphorylation of numerous substrates, resulting in the alteration of gene expression programs [36]. Suppression of JNK signaling results in the downregulation of Notch1, which is crucial to regulating self-renewal and determining the fate of mammary stem cells [36,37]. Our data demonstrated that 4-BR and the JNK inhibitor inhibited Notch1 expression, suggesting that 4-BR may contribute to JNK-mediated gastric cancer stemness regulation.

In this study, we performed a functional and molecular characterization of human gastric CSCs, and demonstrated the cancer stemness inhibition by 4-BR by using different functional approaches and stem-cell-related markers characterization, including stem cell surface markers, CD24 and LGR5; and stemness-related transcription factors, SOX2, NANOG, Oct-4, and detoxifying enzyme, ALDH1. Our results provided evidence that 4-BR not only inhibited gastric CSC stemness markers through SIRT3-JNK-mediated pathway but also enhanced chemosensitivity to 5-FU.

There is an increasing awareness of the importance of natural products for human health. Dietary phytochemicals are the candidates for anticancer research, and represent an important strategy to target CSCs. Natural products are a source of bioactive compounds. The 4-BR analog, resveratrol, which is primarily found in red grape, was also reported to enhance the chemotherapeutic response and reverse the cancer stemness in several cancer types [52,53]. Unlike 4-BR modulating SIRT3, resveratrol stimulates SIRT1 activity and expression to inhibit cancer stemness [54]. The different cancer stemness inhibition mechanisms of these natural compounds may be introduced into potential strategies in personal precision medicine in cancer therapy.

## 5. Conclusions

We demonstrated the ability of 4-BR to reduce cancer stemness and chemoresistance in gastric cancer cells. 4-BR may utilize the SIRT3-JNK-mediated pathway to downregulate gastric cancer stemness and increase chemosensitivity. 4-BR is a promising candidate for use in clinical chemotherapy.

## Data Availability

All data sets generated or analyzed in this study were included in the published article. Detailed data sets supporting the current study are available from the corresponding author upon request. This study did not generate new codes.

## Sample Availability

Samples of the compounds are available from the authors.
